# Toxic Hepatitis Associated With the Use of Contraceptives: A Diagnosis to Bear in Mind

**DOI:** 10.7759/cureus.21220

**Published:** 2022-01-13

**Authors:** Alejandro Blanquicett, Maria Cristina Martinez-Ávila, Zulay Mondol, Pedro Imbeth Acosta

**Affiliations:** 1 Gastroenterology and Hepatology, Corporación Universitaria Rafael Nuñez, Cartagena, COL; 2 Intensive Care Unit, Nuevo Hospital Bocagrande, Cartagena, COL; 3 Internal Medicine, Universidad del Sinu, Cartagena, COL; 4 Gastroenterology and Hepatology, Nuevo Hospital Bocagrande, Cartagena, COL

**Keywords:** drug-induced acute liver failure, combined injectable contraceptives, use of contraceptives, cholestasis, severe hepatotoxicity

## Abstract

Drug-induced liver injury (DILI) is a rare condition with a high burden of morbidity and risk of severe complications that may even require liver transplantation to survive. There are no pathognomonic diagnostic tests for this condition, thus being a challenge it is considered a diagnosis of exclusion. We present the case of a patient who, after starting intramuscular contraceptives, presented with acute alteration of liver function that requires the ruling out of multiple pathologies that could be the cause of the biochemical findings. With the pharmacological suspension and initiation of support measures, full recovery was achieved.

## Introduction

Drug-induced liver injury (DILI) is a rare condition, present in <1% of patients with diverse symptoms [1]; however, it carries high morbidity and mortality, remaining the leading cause of acute liver failure in the United States [2]. There are currently more than 1000 drugs and natural products that can cause liver compromise and severe complications, which sometimes require transplantation to survive [1]. Despite this, its diagnosis continues to be a challenge for gastroenterologists.

This entity is recognized as an alteration in liver function tests with or without associated clinical symptoms. To date, there are no gold standard diagnostic tests or pathognomonic histological changes, requiring the exclusion of other more frequent etiologies of liver damage before confirming this disease. The RUCAM (Roussel Uclaf Causality Assessment Method) update uses well-defined cut-off points to identify liver injury, with alanine aminotransferase (ALT)> 5 times the upper limit of the normal range and an alkaline phosphatase> 2 times the upper limit of the normal range [3]. Any alteration that meets these criteria requires a thorough evaluation of the patient. The diagnosis of DILI being an entity of exclusion requires ruling out multiple pathologies that could be the cause of the biochemical findings.

Herein, we present a case of a female patient who developed abnormalities in liver function tests after starting contraceptives for the management of dysfunctional uterine bleeding, many etiologies were ruled out and damage was attributed to the use of combined injectable contraceptives (CIC) (dihydroxyprogesterone acetophenide, 150mg + ethinylestradiol, 10mg). The importance of knowing these alterations is highlighted in order to find a timely diagnosis and treatment.

## Case presentation

We present the case of a 38-year-old female patient with a history of abnormal uterine bleeding with secondary anemia that required management with a combined injectable contraceptive (CIC) (dihydroxyprogesterone acetophenide, 150mg + ethinylestradiol, 10mg) monthly, without other exposures or toxic habits. She presented to the clinic for jaundice syndrome that started 15 days after applying CIC. The rest of the physical examination was unremarkable.

Blood tests were performed to obtain the following results: hemoglobin (Hb) 10.7mg/dL; mean corpuscular volume (MCV) 85; platelets 372,000/mcL; alanine transaminase (ALT) 496 U/L; aspartate aminotransferase (AST) 366 U/L; alkaline phosphatase (ALP) 1132 U/L; total bilirubin 2.8 mg/dl; direct bilirubin 1.65 mg/dl; indirect bilirubin 0.43 mg/dl; lactate dehydrogenase (LDH) 3110 U/L; gamma-glutamyl transferase (GGT) 1439 U/L, evidencing hepatitis with a clear predominance of the cholestatic pattern; however, the origin of hepatitis was yet to be determined.

Further, in this case, R-value was calculated to be 1.26 pointing to a cholestatic injury in the context of a patient who's receiving CIC for the first time, so medication was suspended and supportive management was started. The R-value is used to define whether the hepatic injury is “hepatocellular”, “mixed”, or “cholestatic.” It is calculated by dividing the alanine aminotransferase (ALT) value by the alkaline phosphatase (ALP), using multiples of the upper limit of the normal range for both values. R ratios of >5 define a hepatocellular, <2 a cholestatic, and between 2 and 5 a mixed pattern of enzymes. Also, complementary tests that included metabolic, autoimmune, virological, neoplasia, partial thromboplastin time (PTT), prothrombin time (PT), fibrinogen, thyroid stimulation hormone (TSH), antinuclear, antimitochondrial, anti-smooth muscle, anti-liver-kidney microsomal (LKM) antibody, ceruloplasmin, and sideremia antibodies to rule out the cause of liver injury were performed and results for all of these indicated normal levels. Tests for hepatitis A, B, and C serology, HIV, leptospira, dengue, Epstein-Barr, herpes simplex virus I and II, cytomegalovirus, and mononucleosis were negative. Additionally, the alpha-fetoprotein values, alpha-1-antitrypsin, and tumor markers were negative. Abdominal ultrasound and cholangioresonance were normal, intra- and extrahepatic non-dilated bile duct were without alterations, and the pancreas was also normal. Other findings were of no significance. 

Under the suspected diagnosis of toxic liver disease induced by CIC, a Council for International Organizations of Medical Sciences (CIOMS)/RUCAM score [3] of 9 points was estimated with a highly probable prediction. Supportive management was continued with daily biochemical monitoring. After one month, she presented progressive normalization of parameters, clinically she was asymptomatic and was discharged from the hospital. A liver biopsy was not performed due to a favorable evolution. In the follow-up examinations three months after the event she remained asymptomatic with a complete favorable resolution. Abdominal ultrasound revealed no pathological changes.

## Discussion

Contraceptives have been associated with various liver diseases such as cholestasis, adenomas, Budd-Chiari syndrome, cholelithiasis, hepatocellular carcinomas, dilatation of sinusoid peripherals, among others [4]. It usually begins weeks to months after the administration of estrogen and progestin steroid CICs, causing intrahepatic cholestasis with pruritus and jaundice [4]. These hepatotoxic reactions are infrequent processes, but they can lead to high morbidity and even mortality [4]. 

The combination of dihydroxyprogesterone acetophenide and ethinylestradiol was used for the first time in humans as a contraceptive in 1964, being a long-acting CIC that stimulates its deposit in adipose tissue from where it is released progressively, maintaining therapeutic concentrations that allow monthly administration [5]. It is metabolized through the liver by cytochrome p450. Estradiol reaches peak concentrations three to six days after its administration; for its part, hydroxyprogesterone has a half-life of 24 days. The metabolites are eliminated in bile and feces; one part undergoes enterohepatic circulation [5].

DILI is a multifaceted phenomenon that can include any pattern of liver damage, in people with acute or chronic exposure. Although cases of DILI secondary to the use of progestins have been documented, this damage usually occurs in the first weeks of use, the most classic damage being the hepatocellular pattern, the alterations are more commonly seen in association with high doses of estrogens. The damage usually reverses after dose modification or suspension. New estrogen formulations are not usually associated with alterations in liver function tests, if they do, the most common presentation is with cholestatic patterns manifested in the first months of use and rarely after six months. Fatal events have not been documented with the use of progestins and estrogens; however, DILI secondary to the use of these is a condition to consider within the differential diagnosis for addressing abnormal liver function tests.

The presentation is usually insidious manifesting as fatigue, itching, nausea, choluria, and jaundice [1]. In addition, as noted in various reviews, exudative cholestatic reactions are sometimes associated with hepatocellular necrosis and hepatic lobular inflammation, which can simulate a case of acute viral hepatitis [6], as occurred in the present case. The CIOMS/RUCAM [3] score was used correctly in this case, finding a highly probable prediction. Given these findings, the pharmacological suspension was indicated, and the patient was given support measures after which she presented a favorable evolution. The approach to which the patient underwent is adequate, ruling out various pathologies that could explain alterations in liver function tests, supporting then the diagnosis of DILI. There was no liver biopsy done, however, in this case, it was not considered essential due to the clinical course presented by the patient.

## Conclusions

The idiosyncratic nature of DILI forces us to know and document the timing, dose, and indication of the drugs used by our patients. The presentation of this case emphasizes the importance of correlating biochemical alterations with the patient's medical history, documenting the correct elimination of differential diagnoses, and the use of tools such as RUCAM to support the diagnosis of DILI. It is important to know this potentially serious pathology and that the free sale of CIC and its more frequent use is leading to an increase in the appearance of DILI.
